# Fear of childbirth: prevalence and associated factors in pregnant women of a maternity hospital in southern Brazil

**DOI:** 10.1186/s12884-023-05948-0

**Published:** 2023-09-02

**Authors:** Ana Paula Maia Dal Moro, Gabriella Soecki, Fernanda Schier de Fraga, Ricardo Rasmussen Petterle, Sarah Zanghellini Rückl

**Affiliations:** 1 Medicine Student, Federal University of Paraná, Curitiba, Paraná Brazil; 2 Departament of Tocogynecology, Federal University of Paraná, Curitiba, Paraná Brazil; 3 Departament of Integrated Medicine, Federal University of Paraná, Curitiba, Paraná Brazil; 4 Departament of Psychiatry and Forensic Medicine, Federal University of Paraná, Curitiba, Paraná Brazil

**Keywords:** Maternal mental health, Pregnancy, Fear of childbirth, Prenatal care, Tocophobia

## Abstract

**Background:**

The fear of childbirth (FOC) harms maternal and fetal health, however it has been little studied in Brazil. This research aimed to determine the prevalence of FOC in a maternity hospital in southern Brazil and identify its associated factors.

**Methods:**

The Wijma Delivery Expectancy Questionnaire – W-DEQ(A) was used to assess the prevalence of FOC, and its relationship with sociodemographic variables, gestational history, aspects of the current pregnancy, knowledge about childbirth, anxiety symptoms (Beck Anxiety Inventory), depressive symptoms (Edinburgh Postnatal Depression Scale), and perception of social support (Multidimensional Scale of Perceived Social Support) was investigated. Questionnaires about the content of FOC and information sources regarding childbirth were also applied.

**Results:**

We interviewed 125 pregnant women between 28 and 36 weeks of pregnancy between July and September of 2021, and 12% of them scored ≥ 85 on the W-DEQ(A), indicating severe FOC. There was a significant correlation between FOC and anxiety symptoms (*r* = 0.50, *p* < 0.001), depressive symptoms (*r* = 0.34, *p* < 0.001), and poor social support (*r* = -0.23, *p* = 0.008). FOC was lower in pregnant women with complete elementary education when compared to those with higher education (*p* = 0.003), however, those with negative experiences in previous deliveries had more FOC than those who had had positive experiences (*p* = 0.001). More than 85% of them fear fetal distress.

**Conclusions:**

FOC is a prevalent condition that impacts the mental health of pregnant women. Therefore, health professionals should recognize and address it during prenatal care to provide integral maternal–fetal care and improve the childbirth experience.

**Supplementary Information:**

The online version contains supplementary material available at 10.1186/s12884-023-05948-0.

## Background

For women who become mothers, childbirth is a remarkable experience, permeated by expectations and changes that can generate concern and insecurity [1]. Therefore, fear of childbirth (FOC) tends to be present during pregnancy, however, at higher levels, it impairs the quality of life of pregnant women [2]. When FOC becomes severe and an avoidant attitude towards childbirth is undertaken, it is called tokophobia, which can be classified as primary, when it occurs in nulliparous women, or secondary, in multiparous women, due to negative experiences in previous births [3, 4].

The content of the fear is diverse and includes physiological, psychological, and social aspects related to childbirth, These aspects include maternal–fetal risk, pain, a lack of emotional capacity to experience childbirth, medical interventions, body changes during or after childbirth, and a lack of economic or social support [5–8].

The W-DEQ-The Wijma Delivery Expectancy/Experience Questionnaire was the first and most used tool to rate FOC during pregnancy [9, 10]. It is divided into versions A and B. The W-DEQ(A) assesses the cognitive antepartum expectations, and the WDEQ(B) evaluates the postpartum experience [2, 4, 10]. It has an α-Cronbach of 0.93 and has not yet been validated for Brazilian Portuguese [6, 9].

Another well-studied tool, more promising for clinical use than research, is the FOBS-Fear of Birth Scale. It consists of the question “How do you feel right now about the approaching birth?” and two responses with the extremes “calm/worried” and “no fear/intense fear” [2, 10–13]. Other developed tools that are less used are the Delivery Fear Scale (DFS) and the Childbirth Attitudes Questionnaire (CAQ) [14, 15].

A study conducted with 7200 pregnant European women between 2008 and 2010 evaluated the prevalence of FOC using the W-DEQ(A). The mean prevalence of FOC was 11%, but there was significant variation between the countries evaluated. For nulliparous women, this variation ranged from 4.5% to 15.6% and, for multiparous women, from 7.6% to 15.2% [16, 17]. Nilsson et al. [2], in a systematic review, showed that the global prevalence of FOC is between 6.3% and 14.8%.

Some pregnant women are more prone to FOC than others. Ryding et al. [18] demonstrated the associations between personality traits and FOC: women with higher socialization tend to feel less fear, whereas those with somatic anxiety, irritability, impulsivity, and distrust have more fear. In addition, other factors associated with FOC have already been described: presence of depressive and/or anxious symptoms, lack of social support, negative experience in previous childbirth, preference for elective cesarean section, and others [19–21].

One of the negative implications of FOC is the need for more analgesia during labor [22, 23]. In this regard, Hurtado et al. [24] demonstrated that fear and anxiety during childbirth lead to muscle contraction, which may hinder pelvic dilatation during vaginal delivery and worsen pain, resulting in a cycle of fear, tension, and pain.

In terms of interventions for FOC management, some studies have shown positive outcomes. Hosseini, Nazarzadeh, and Jahanfar [1] demonstrated that giving quality information and providing psychological support to pregnant women during prenatal care can relieve FOC, in addition to reducing the request for cesarean without medical indication, as well as the experience of pain during childbirth [25, 26]. During labor, breathing exercises, massages, and a safe environment can help normalize physiological reactions and promote the release of endorphins, reducing fear and pain [27].

Therefore, this study aims to assess the prevalence of FOC in pregnant women in a maternity hospital in southern Brazil using the W-DEQ(A) scale and identify its associated factors. The content of the fears of pregnant women and their sources of information about childbirth were also investigated. It is expected that the results will optimize the approach of FOC during prenatal care by demystifying beliefs and conviction limitations.

## Methods

### Participants and procedures

This cross-sectional study was conducted in a maternity hospital of the public health system that preferentially attends high-risk pregnancies, located in the state of Paraná in Brazil. For data collection, printed self-administered questionnaires in Brazilian Portuguese were used: (a) WDEQ(A) [9], (b) Beck Anxiety Inventory (BAI) [28–31], (c) Edinburgh Postnatal Depression Scale (EPDS) [32, 33], (d) Multidimensional Scale of Perceived Social Support (MSPSS) [34, 35] and (e) the Psychosocial Profile Assessment (PPA). The inclusion criteria were: Brazilian pregnant women over 18 years of age and between the 28th and 36th week of gestational age, undergoing prenatal care at a high-risk maternity hospital, and able to read and understand written and spoken Portuguese. The third trimester of pregnancy was adopted because previous studies were also carried out at this time of pregnancy, which is the closest to the delivery date. The participants were invited to participate in this study while waiting for their prenatal or emergency appointments at the Maternity hospital, as well as in the inpatient units, between July and September 2021.

**Fig. 1:**
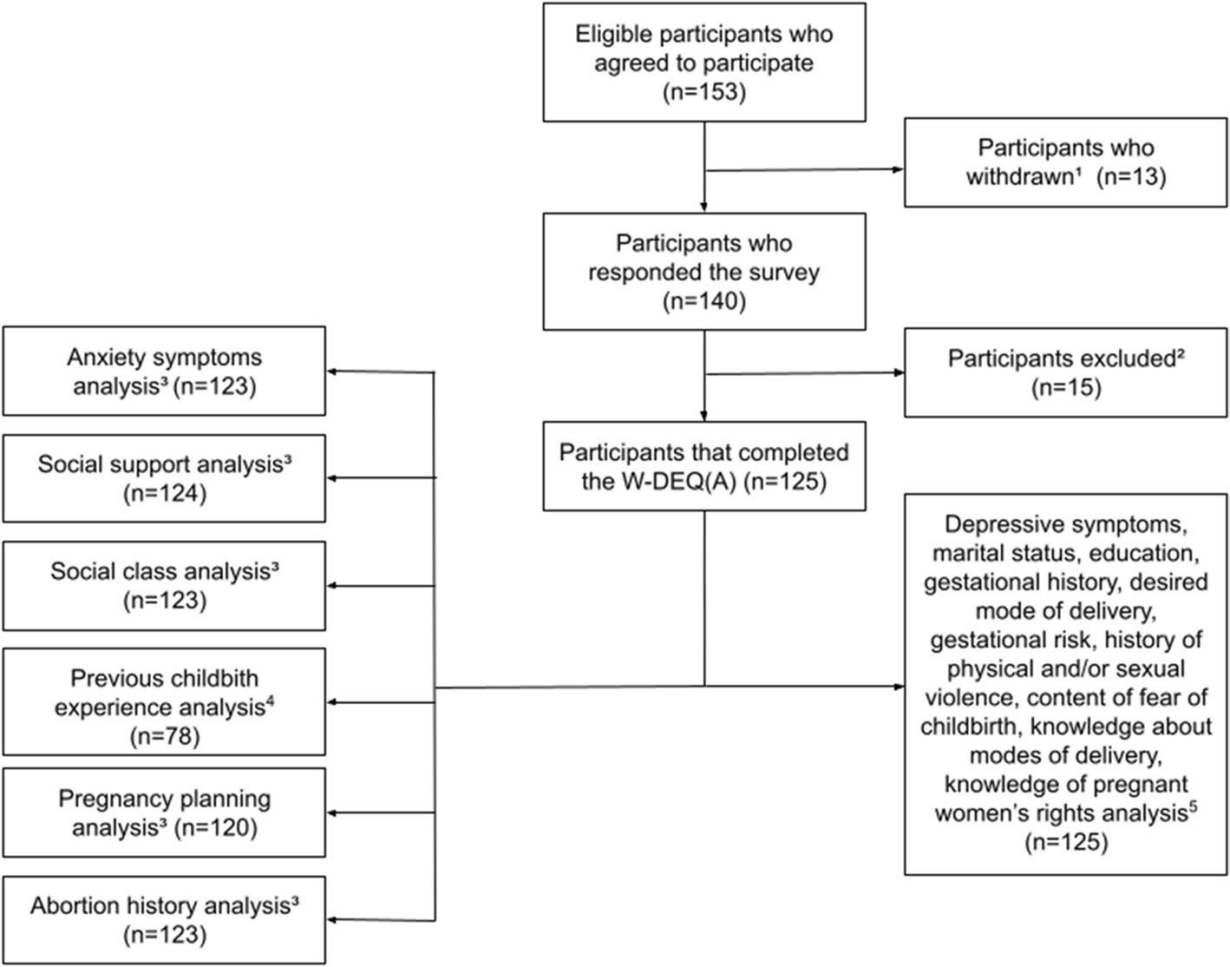
Flowchart for the Study Population ^1^153 eligible pregnant women accepted to participate in the research; of these, 13 withdrew. ^2^Among the 140 pregnant women who responded to the survey, 15 were excluded for leaving the W-DEQ(A) scale incomplete. ^3^Among the 125 participants who completed the W-DEQ(A) scale, 12 were excluded from analyses of specific variables because they left incomplete data. Analysis of anxiety symptoms (*n* = 123), social support (*n* = 124), social class (*n* = 123), pregnancy planning (*n* = 120), and abortion history (*n* = 123). ^4^Among the 125 participants who completed the W-DEQ(A) scale, 79 were multiparous, and 78 answered the question about previous childbirth experience. ^5^Other topics (depressive symptoms, marital status, education, gestational history, desired mode of delivery, gestational risk, history of physical and/or sexual violence, content of fear of childbirth, knowledge about modes of delivery, and knowledge of pregnant women's rights) had no incomplete data and excluded participants (*n* = 125). Created with Google Drawings

To calculate the sample size, a confidence level of 95% was adopted with a margin of error of 6%, and, based on previous studies, an estimated prevalence of 11% was expected [2, 16, 17]. Thus, the sample size should have at least 105 participants. The sample selection was made in a non-probabilistic way. During collection days, all scheduled patients who met the inclusion criteria were invited to participate. One hundred and fifty-three pregnant women were recruited, and 28 were excluded, 13 for dropping out of the study and 15 for leaving the W-DEQ(A) incomplete. Participants who left other randomly missing items were not excluded, and only the variable in question was disregarded from their specific analysis, as shown in Fig. 1. Among the 125 pregnant women included, 109 were approached while waiting for prenatal appointments, three in the emergency room, and 13 were hospitalized in the inpatient unit. After each day of collection, the collaborators referred pregnant women with positive screening for FOC, depression, or anxiety to the Psychiatry outpatient clinic.

### Measures

#### Demographic data

The PPA was created by the authors to identify sociodemographic data and psychosocial aspects of the participants. The sociodemographic characteristics collected were: social class according to the New Brazilian Socioeconomic Classification Criteria [36], education, marital status, and history of sexual and/or physical violence. The age of the participants and the gestational age were identified in the chart.

#### Fear of childbirth

To measure FOC the 33-item W-DEQ(A) was adopted [9]. Responses are distributed on a Likert scale, with the extremes being zero to “never/not at all” and five to “very often/extremely”. Values ≥ 85 were considered “intense FOC” and ≥ 100, “very intense FOC” or “tokophobia” [2, 10]. No study known to the authors applied this scale to Brazilian pregnant women. The translation of the scale was carried out by the researchers, one of whom translated it from English to Portuguese, and then another translated it from Portuguese to English. The English version was then compared to the original. Internal consistency was calculated using α-Cronbach.

In the PPA, the FOC content was evaluated by the question “Are you afraid of?”, listed for 13 FOC aspects with dichotomous answers (yes or no).

#### Obstetric history

The obstetric history was addressed in three PPA items: parity, previous childbirth experience(s), and abortion history.

#### Current pregnancy

The current pregnancy was addressed in three PPA items: pregnancy planning, desired mode of delivery, and gestational risk.

#### Knowledge about childbirth

Two aspects of childbirth knowledge were assessed by the PPA. First, the two main sources of information used by pregnant women to acquire knowledge about delivery methods. Second, the participants' level of knowledge about childbirth is assessed through five statements based on Brazilian laws that protect pregnant women and address the rights of parturients, with dichotomous answers (true or false) [37]. The median value of correct answers was adopted as a cutoff point to determine whether the level of knowledge was adequate or not.

#### Anxiety symptoms

The Beck Anxiety Inventory (BAI) scale, consisting of 21 items, assessed anxiety symptoms [28]. Each item has a Likert scale ranging from zero, which means "not at all" to three, which means "severely". The total score ranges from zero to 63, and the higher the score, the more symptoms the woman has. We performed the validated Brazilian version [29–31]. The classification of anxiety levels adopted was: minimal symptoms (0–10), mild (11–19), moderate (20–30) and severe (31–63) [31].

#### Depressive symptoms

Depressive symptoms were measured by the Edinburgh Postnatal Depression Scale (EPDS), which is also valid for investigation in pregnancy [32, 33]. It has ten questions, whose answers are distributed into four Likert points regarding the frequency of symptoms. The total score ranges from zero to 30. The version validated in Portuguese by Santos et al. [33] was used, considering a cut-off point ≥ 10 [38].

#### Perceived social support

Perceived social support was measured using the Multidimensional Scale of Perceived Social Support questionnaire (MSPSS), validated for use in Brazil, which assesses the support received from friends, family, and partners [34, 35]. It has 12 statements with Likert responses ranging from one (very strongly disagree) to seven (very strongly agree), with a total score ranging from one to seven. Values between 1 and 2.9 are considered low social support, between 3 and 5, moderate support, and between 5.1 and 7, high support [39].

### Statistical analysis

For categorical variables, absolute and relative frequencies were calculated, while for numerical variables, medians and minimum and maximum values (min–max) were calculated. In this study, statistical significance was adopted at *p* < 0.05. Data normality was verified by the Shapiro–Wilk test. Thus, the non-parametric Mann–Whitney, Spearman, and Kruskal–Wallis correlation tests were applied, followed by a multiple comparison test with Bonferroni correction. Finally, data analysis was performed using the statistical software R [40], version 4.1.2.

## Results

### Sociodemographic data

The sociodemographic data are described in Table 1. The age of the participants ranged from 18 to 48 years, with a mean of 29.25 years. Most of them were married (74.4%), 60% studied up to high school, and 88.6% belonged to classes B or C.

**Table 1:**
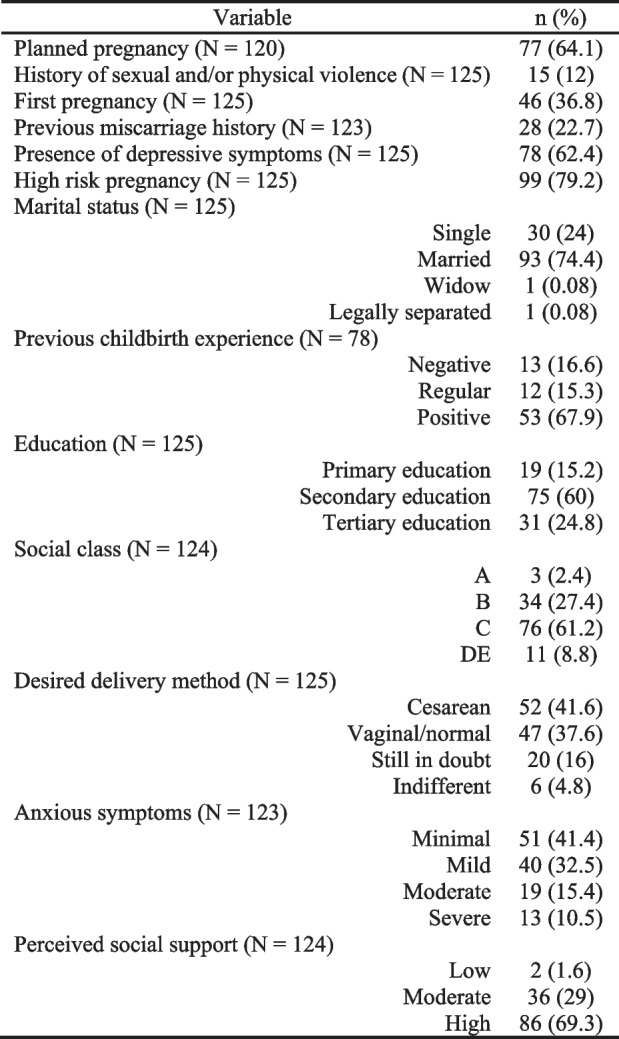
Sample characteristics

### Fear of childbirth

The mean score obtained in the W-DEQ(A) was 57.1, and 12% of the participants scored ≥ 85. Of these, 6.4% scored between 85 and 99 points, indicating intense FOC, while 5.6% reached values ≥ 100, indicating very intense FOC or tokophobia. The α-Cronbach of the version translated by the authors was 0.92 (95% CI = 0.896–0.937).

There was no statistical difference in the W-DEQ(A) score in relation to age, marital status, social class, and history of sexual and/or physical violence—Tables 2 and 3. Regarding education, there was a significant difference, in the Kruskal–Wallis test (*p* = 0.013) and, later, in the multiple comparisons test, between the scores obtained by pregnant women with Primary education (median = 38 min–max = 6–102) compared to those with Tertiary education (median = 69 min–max = 16–136 *p* = 0.009)—Table 3.

**Table 2:**
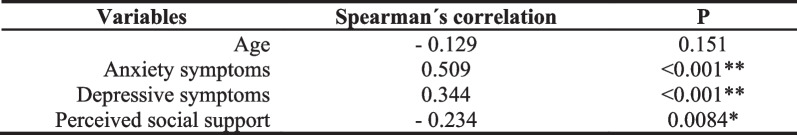
Correlation between W-DEQ(A) total score and age, anxiety symptoms, depressive symptoms, and perceived social support

^*^p ≤ 0.01; ** *p* ≤ 0.001

**Table 3:**
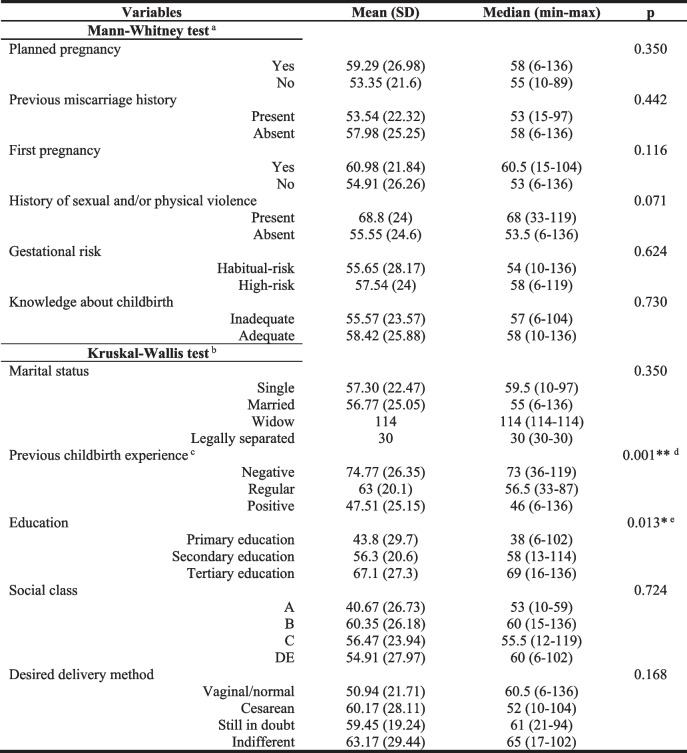
Correlation between W-DEQ(A) scores and sociodemographic, gestacional, and psychosocial variables

*SD* Standard deviation, *min* minimum value, *max* maximum value

^*^p ≤ 0.01; ** *p* ≤ 0.001

^a^ For dichotomous variables, the Mann–Whitney test was applied

^b^ For qualitative variables, the Kruskall-Wallis test was applied

^c^ The analysis on previous childbirth experience just involved the multiparas (*N* = 78)

^d^ In the multiple comparison test, there was a significant difference when negative experiences were compared with positive ones (*p* = 0.002)

^e^ In the multiple comparison test, there was a significant difference when Primary education was compared with Tertiary education (*p* = 0.009)

Table 4 shows FOC´s content. The most marked statements were: "that my child suffers in some way" (85.48%), "being attended by a rude medical team" (83.87%), and "having a very long delivery" (82.25%).

**Table 4:**
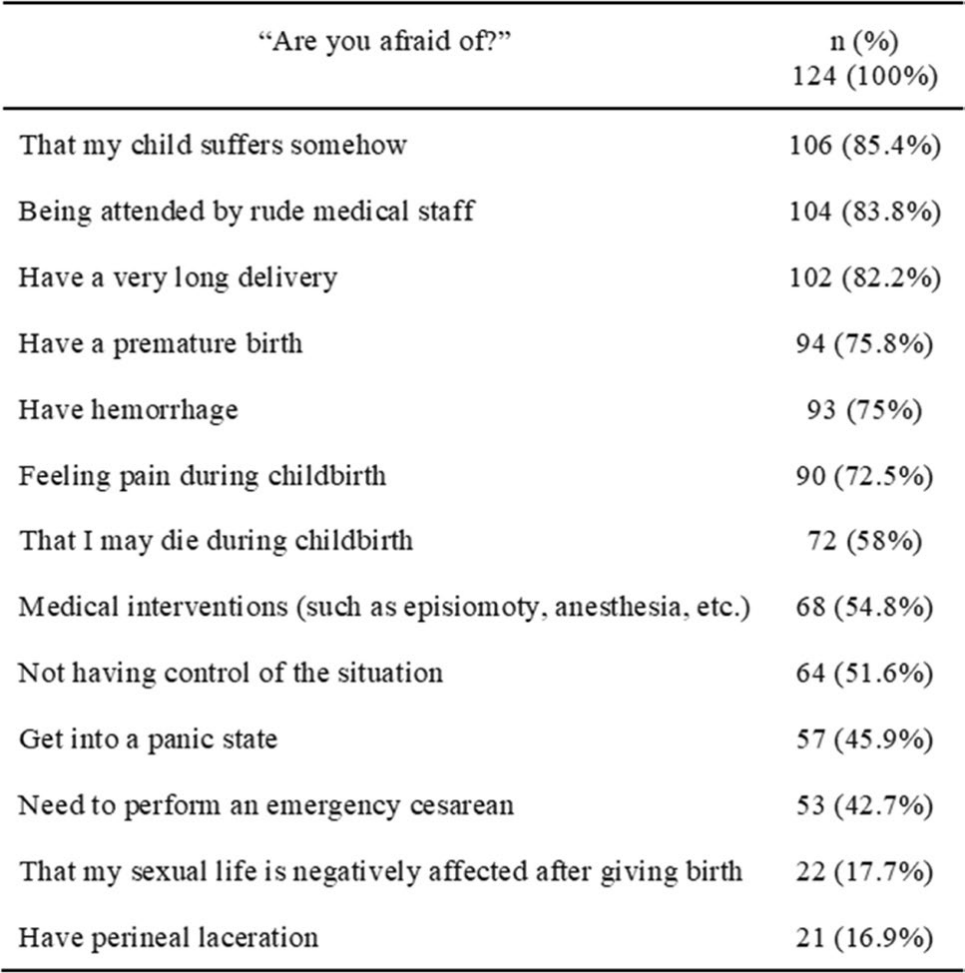
Fear of childbirth's content: affirmative answers

### Fear of childbirth and obstetric history

63.2% of the participants were multiparous, of whom 67.9% classified their previous childbirth experiences as positive, 15.4% as regular, 16.7% as negative, and 22.8% had a history of abortion. There was no statistical difference in the W-DEQ(A) score in relation to parity and abortion history—Table 3. Regarding the experience of previous births, a significant difference was observed, using the Kruskal–Wallis test (*p* = 0.001) and, later, the multiple comparisons test, between the scores of pregnant women with negative experiences (median = 73 min–max = 36–119) compared to those with positive experiences (median = 46 min–max = 6–136 *p* = 0.002)—Table 3.

### Fear of childbirth and current pregnancy

Most pregnant women had planned the pregnancy (64.1%) and were at high risk (79.2%). Regarding to the mode of delivery, 37.6% wanted to perform a vaginal delivery and 41.6% wanted a cesarean. There was no statistical difference in the W-DEQ(A) score in relation to pregnancy planning, desired mode of delivery, and gestational risk—Table 3.

### Knowledge about childbirth

**Fig. 2:**
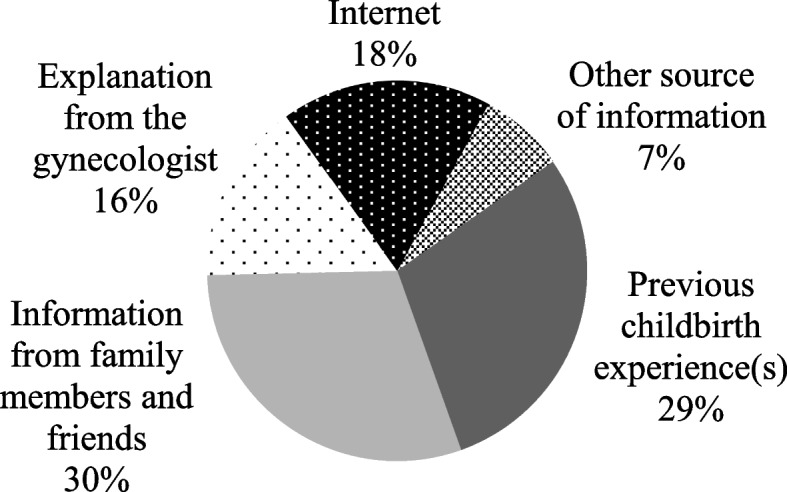
Main source of knowledge about delivery methods. 30% of pregnant women reported that their knowledge about the delivery methods (vaginal, normal, or cesarean) was acquired in the first place through information from family members and friends. 29% through previous childbirth experience(s). 16% with an explanation from the gynecologist. 18% internet. 7% other sources of information Created with MSOffice

**Fig. 3:**
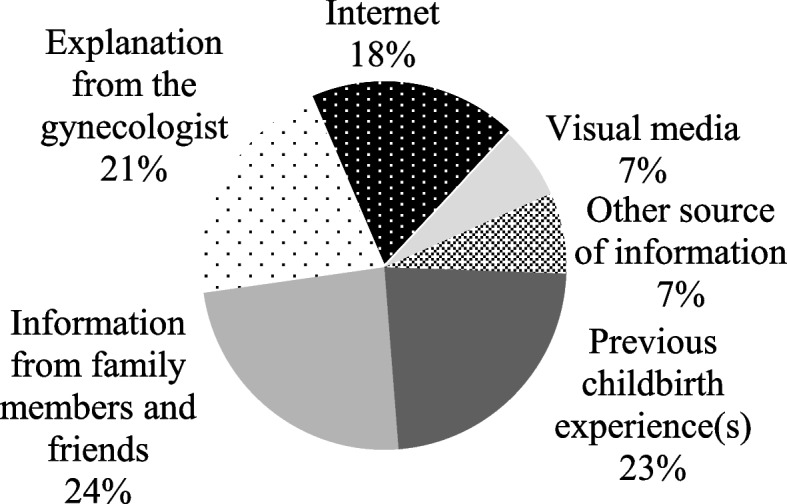
Second main source of knowledge about delivery methods. 24% of pregnant women reported that their knowledge about the delivery methods (vaginal, normal, or cesarean) was acquired, secondly, through information from family members and friends. 23% through previous childbirth experience(s). 21%with an explanation from the gynecologist. 18% internet. 7% visual media. 7% other sources of information Created with MSOffice

Figures 2 and 3 show, respectively, the first and second main sources of information about the delivery methods. Assessing the level of knowledge about childbirth, the statements with the fewest correct answers referred to episiotomy (60%) and Kristeller's maneuver (52.8%)—Table 5. There was no significant difference between adequate (4–5 correct answers) or inadequate (0–3 correct answers) knowledge about childbirth and FOC—Table 3. Four correct answers were adopted as cutoff points for adequate knowledge because they were the median value of correct answers.

**Table 5:**
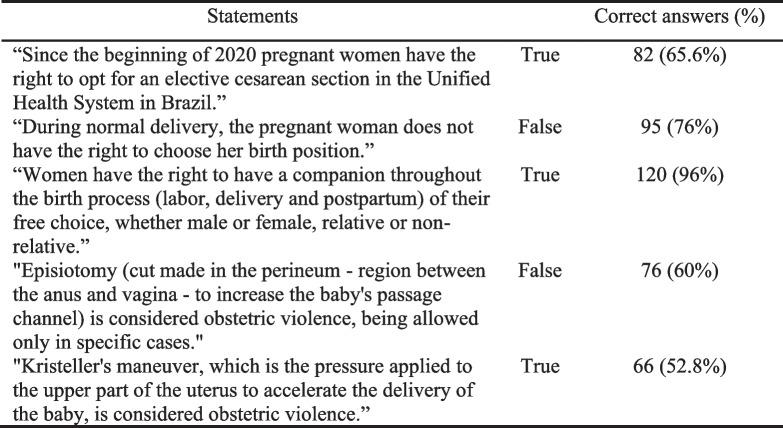
Knowledge about childbirth and rights of parturients

### Fear of childbirth and anxiety

The average BAI score was 15.3. Among the participants, 25.9% had moderate or severe anxiety symptoms. There was a moderately positive correlation between anxiety symptoms and FOC (r = 0.5, p < 0.001)—Table 2.

### Fear of childbirth and depressive symptoms

The mean EPDS score was 9.2, and 37.6% reached the cutoff ≥ 10. There was a weak positive correlation between depressive symptoms and FOC (*r* = 0.34, *p* < 0.001)—Table 2.

### Fear of childbirth and perception of social support

The average MSPSS score was 5.64. Among the participants, 69.3% had high social support. There was a weak negative correlation between perceived social support and FOC (r = -0.23, *p* = 0.008)—Table 2.

## Discussion

In this study, data collected through self-administered questionnaires was used to assess the prevalence of FOC and its associated factors in Brazilian pregnant women. The prevalence of intense fear of childbirth (W-DEQ(A) ≥ 85), including tokophobia (W-DEQ(A) ≥ 100) was 12%, similar to the findings of Lukasse et al. [16], Nilsson et al. [2] and O'Connell et al. [41]. The internal consistency of the W-DEQ(A) translated by the researchers was adequate (α-Cronbach = 0.92) and very close to that of the original version (α-Cronbach = 0.93) [9].

Although only 12% of the participants had an intense fear of childbirth, when asked about specific fears, they referred to several situations. More than 80% of them confirmed that they were afraid that their child would suffer in some way, such as being attended by a rude medical team or having a very long delivery, reiterating previous studies [42, 43].

The main source of information on delivery modes was the experience of previous deliveries, demonstrating that the experience of previous deliveries influences the expectations of pregnant women regarding the current delivery. Furthermore, pregnant women who had positive experiences in previous deliveries had less FOC (median = 46 min–max = 6–136) compared to those with negative experiences (median = 73 min–max = 36–119 *p* = 0.002), corroborating the results of Dencker et al. [20] and Størksen et al. [44]. The second main source of information was family and friends, evidencing the influence of people close to the pregnant woman, as previous studies suggest [42, 45, 46]. Visual media were the least mentioned option as a source of information. However, qualitative studies highlight that the representation of pregnancy in the media contributes to the construction of beliefs and a mental image of childbirth [47, 48].

As for the pregnant women’s knowledge about childbirth, the two statements about medical interventions (episiotomy and Kristeller's maneuver) had the lowest hit rates. Possibly, this was observed because information of this kind is less addressed during medical appointments. However, knowledge of such information provides protection to pregnant women as it facilitates the recognition of behaviors classified as obstetric violence [49].

There was no significant difference between the pregnant women's level of knowledge about childbirth, measured by the median of correct answers in a "true or false" question, and FOC. However, the difference in schooling among the participants and the objective nature of the assessment adopted may have led to random hits and misses. Therefore, a more adequate alternative would be to adopt a scale of perception of informative support, that also considers the subjective aspects of the participants – whether they feel well informed or not. In a cross-sectional study, O'Connell et al. [50] used a scale of perception of informative support about the puerperium and found a significant association between low support and higher levels of FOC. Therefore, in future studies, the authors suggest the elaboration of a scale of perception of informative support specific to childbirth.

In this study, there was a weak correlation between FOC and depressive symptoms (r = 0.34, *p* < 0.001), as well as between FOC and perception of social support (r = -0.23, *p* = 0.008), and a moderately positive correlation between anxiety symptoms and FOC (r = 0.5, *p* < 0.001), reiterating previous studies [14, 19, 20]. There was also a significant difference between FOC and education (*p* = 0.013), however, unlike previous studies, higher levels of education implied higher FOC [20].

Pregnant women with a history of sexual and/or physical violence had a higher median (median = 68 min–max = 33–119) than those without this history (median = 53.5 min–max = 6–136). However, this difference was not significant (*p* = 0.07), differing from previous studies [17, 51, 52]. This divergence probably occurred due to the smaller sample size used in the present study.

There was no significant correlation between FOC and abortion history, age, and marital status, corroborating previous studies [20, 21, 53–55]. There was also no association between pregnancy planning and FOC, unlike Dencker et al. [20].

Quantitatively, the pregnant woman's social class did not influence the FOC, but Roosevelt and Low [43] qualitatively demonstrated that perceptions of race, class, gender, and sexual orientation affect the experience within the health system, especially in periods of greater vulnerability, such as pregnancy.

There was no association between FOC and preference for cesarean delivery, contrary to previous studies [8, 19]. Most pregnant women included in this study were at high obstetric risk (79.2%), having an indication for cesarean section, a fact that may have interfered with the responses, as pregnant women who are confident that they will undergo cesarean section may have their FOC “masked” and not detectable by the W-DEQ(A) [14]. There was also no difference between gestational risk and FOC. This result agrees with the study by Mohamamdirizi, Mohamadirizi, and Mohamadirizi [56] and diverges from Ben-Ari, Chasson, and Abu-Sharkia [57].

Evidence regarding the association between parity and FOC is contradictory [11, 22, 58]. In this study, it was not observed. Dencker et al. [20] suggest that nulliparous and multiparous women have similar levels of fear, but for different reasons, and that it would be more appropriate to differentiate “fear of childbirth” from “fear after childbirth”. This corroborates the concepts of primary and secondary tokophobia, and agrees with the distinction between WDEQ(A) and W-DEQ(B).

The sample of this study is representative of a southern Brazilian maternity, but not of the Brazilian population. Brazil is a large country in terms of territory and sociocultural diversity, which interferes with the psychosocial aspects related to FOC. Therefore, in the future, it is important that larger samples be adopted, including pregnant women from different regions of the country, both in the public and private health systems.

This study was carried out in Paraná, which is the only state in Brazil that allows women to choose their mode of delivery. In January 2020 [59], the law nº 9.394/1996, nº 20,127 was approved in the state, providing for the right of women to choose between vaginal delivery and cesarean delivery. Until then, the public health system only released elective cesareans in cases of fetal or maternal risk. Otherwise, delivery should be vaginal. This context may influence the results of the present study.

Data collection was conducted during the COVID-19 pandemic. A study conducted with Polish pregnant women showed that fear of COVID-19 was a significant mediator in the relationship between stress and FOC [60]. Another study involving Italian pregnant women showed that many of them were afraid that Sars-CoV-2 could induce fetal structural anomalies, fetal growth restrictions, or premature birth and demonstrated, prospectively, that the lockdown led to a significant increase in the levels of anxiety of pregnant women [61]. Taubman – Ben-Ari, Chasson, and Abu-Sharkia [57] also highlighted that social isolation weakened the pregnant woman's support network. Furthermore, Brazilian qualitative studies have suggested that the prohibition of a companion during childbirth and the absolute restriction of visitors may have generated feelings such as fear and anxiety [62, 63].

Although there is evidence regarding the impact of the pandemic on the mental health of pregnant women, it is not possible to conclude whether this context promoted an increase in the prevalence of intense FOC detectable by the W-DEQ(A), since the results obtained here were very close to those of international studies carried out before the pandemic. Therefore, conducting studies that evaluate the FOC of Brazilian pregnant women after the pandemic will help to clarify this question.

The strength of this study was the translation of a tool that measures FOC, the W-DEQ(A), and exhibits adequate internal consistency. Additionally, this study was a pioneer in researching FOC in Brazilian pregnant women. The primary limitations of this study were the small sample size and the COVID-19 pandemic. The high percentage of high-risk pregnant women included in this study may also have influenced some results. Finally, the wide sociocultural diversity of Brazil does not allow for the generalization of the results presented here to other regions of the country.

## Conclusion

The pregnant women in this study had FOC detectable by the W-DEQ(A), including tocophobia. The content of FOC is diverse and can involve fear of fetal distress, rude medical staff, and the duration of delivery. Anxiety symptoms, depressive symptoms, social support, and education level were associated with FOC. Experiences from previous pregnancies, as well as information obtained from family members and friends, were important sources of knowledge and influenced expectations regarding childbirth. FOC is still little researched in Brazil. Therefore, the results presented here highlight the need to discuss this issue during antenatal appointments, contributing to high-standard maternal–fetal care.

### Supplementary Information

**Addition file 1:** **Table 1.** The Wijma Delivery Expectancy/Experience Questionnaire (W-DEQ) version A translated into Portuguese (Brazil) by the authors. **Table 2.** The Wijma Delivery Expectancy/Experience Questionnaire (W-DEQ) version A © 1996 K. Wijma & B. Wijma.

## Data Availability

The datasets generated and analyzed during the current study are available from the corresponding author on reasonable request.

## Abbreviations

BAI: Beck Anxiety Inventory
CAQ: Childbirth Attitudes Questionnaire
DFS: Delivery Fear Scale
EPDS: Edinburgh Postnatal Depression Scale
FOBS: Fear of Birth Scale
FOC: Fear of Childbirth
MSPSS: Multidimensional Scale of Perceived Social Support
PPA: Psychosocial Profile Assessment
W-DEQ(A): The Wijma Delivery Expectancy Questionnaire (Version A)
W-DEQ(B): The Wijma Delivery Experience Questionnaire (Version B)

## References

[1] Hosseini VM, Nazarzadeh M, Jahanfar S (2018). Interventions for reducing fear of childbirth: a systematic review and meta-analysis of clinical trials. Women Birth.

[2] Nilsson C, Hessman E, Sjöblom H (2018). Definitions, measurements and prevalence of fear of childbirth: a systematic review. BMC Pregnancy Childbirth.

[3] Hofberg K, Brockington I (2000). Tokophobia: An unreasoning dread of childbirth. a series of 26 cases. B J Psych.

[4] Calderani E, Giardinelli L, Scannerini S (2019). Tocophobia in the DSM-5 era: Outcomes of a new cut-off analysis of the Wijma delivery expectancy/experience questionnaire based on clinical presentation. J Psychosom Res.

[5] Sheen K, Slade P (2018). Examining the content and moderators of women’s fears for giving birth: A meta-synthesis. J Clin Nurs.

[6] Takegata M, Haruna M, Morikawa M, Yonezawa K, Komada M, Severinsson E (2018). Qualitative exploration of fear of childbirth and preferences for mode of birth among Japanese primiparas. Nurs Health Sci.

[7] Hamama-Raz Y, Sommerfeld E, Ken-Dror D, Lacher R, Ben-Ezra M (2017). The Role of Intra-personal and Inter-personal Factors in Fear of Childbirth: A Preliminary Study. Psychiatr Q.

[8] Demšar K, Svetina M, Verdenik I, Tul N, Blickstein I, Velikonja VG (2018). Tokophobia (fear of childbirth): Prevalence and risk factors. J Perinat Med.

[9] Wjma K, Wijma K, Zar M (1998). Psychometric Aspects of the W-DEQ; a New Questionnaire for the Measurement of Fear of Childbirth. J Psychosom Obstet Gynaecol.

[10] Richens Y, Lavender DT, Smith DM (2018). Fear of birth in clinical practice: a structured review of current measurement tools. Sex Reprod Healthc.

[11] Rouhe H, Salmela-Aro K, Halmesmäki E, Saisto T (2009). Fear of childbirth according to parity, gestational age, and obstetric history. BJOG.

[12] Haines H, Pallant JF, Karlström A, Hildingsson I (2011). Cross-cultural comparison of levels of childbirth-related fear in an Australian and Swedish sample. Midwifery.

[13] Haines HM, Pallant JF, Fenwick J (2015). Identifying women who are afraid of giving birth: a comparison of the fear of birth scale with the WDEQ-A in a large Australian cohort. Sex Reprod Healthc.

[14] Zar M, Wijma K, Wijma B (2002). Relations between anxiety disorders and fear of childbirth during late pregnancy. Clin Psychol Psychother.

[15] Khorsandi M, Ghofranipour F, Hidarnia A, Faghihzadeh S, Ghobadzadeh M (2012). The effect of PRECEDE PROCEED model combined with the health belief model and the theory of self-efficacy to increase normal delivery among nulliparous women. Procedia Soc Behav Sci.

[16] Lukasse M, Schroll AM, Ryding EL (2014). Prevalence of emotional, physical and sexual abuse among pregnant women in six European countries. Acta Obstet Gynecol Scand.

[17] Henriksen L, Schei B, Lukasse M (2016). Lifetime sexual violence and childbirth expectations - a Norwegian population based cohort study. Midwifery.

[18] Ryding EL, Wirfelt E, Wängborg IB, Sjögren B, Edman G (2007). Personality and fear of childbirth. Acta Obstet Gynecol Scand.

[19] Poggi L, Goutaudier N, Séjourné N, Chabrol H (2018). When fear of childbirth is pathological: the fear continuum. Matern Child Health J.

[20] Dencker A, Nilsson C, Begley C (2019). Causes and outcomes in studies of fear of childbirth: a systematic review. Women Birth.

[21] Mello RS, Toledo SF, Mendes AB, Melarato CR, Mello DS (2021). Medo Do Parto Em Gestantes. Femina.

[22] Phunyammalee M, Buayaem T, Boriboonhirunsarn D (2019). Fear of childbirth and associated factors among low-risk pregnant women. Obstet Gynaecol.

[23] Aksoy A, Aydin F, Kucur S, Gözükara I (2016). Maternal and fetal Doppler velocimetry in women diagnosed with fear of childbirth. Niger J Clin Pract.

[24] Hurtado F, Donat F, Escrivá P (2003). La mujer ante la experiencia del parto y las estrategias de afrontamiento. Cuad Med Psicosom Psiquiatr Enlace.

[25] Striebich S, Mattern E, Ayerle GM (2018). Support for pregnant women identified with fear of childbirth (FOC)/tokophobia – A systematic review of approaches and interventions. Midwifery.

[26] Junge C, von Soest T, Weidner K, Seidler A, Eberhard-Gran M, Garthus-Niegel S (2018). Labor pain in women with and without severe fear of childbirth: A population-based, longitudinal study. Birth.

[27] Molina-Fernandez I, Rubio L, Roca-Biosca A, Jimenez M, López M, Sirgo A. Ansiedade e medos das gestantes diante do parto: A importância da sua detecção. Rev Port Enferm Saúde Mental. 2015;17–24. 10.1186/ISRCTN14062994.

[28] Beck AT, Brown G, Epstein N, Steer RA (1988). An inventory for measuring clinical anxiety: psychometric properties. J Consult Clin Psychol.

[29] de Lima Osório F, Crippa JAS, Loureiro SR (2011). Further psychometric study of the Beck Anxiety Inventory including factorial analysis and social anxiety disorder screening. Int J Psychiatry Clin Pract.

[30] DeSousa DA, Mordeno AL, Gauer G, Manfro GG, Koller SH (2013). Revisão sistemática de instrumentos para avaliação de ansiedade na população brasileira. Avaliação Psicol.

[31] Cunha JA (2001). Manual da versão em português das escalas Beck.

[32] Cox JL, Holden JM, Sagovsky R (1987). Detection of postnatal depression: development of the 10-item Edinburgh Postnatal Depression scale. B J Psych.

[33] Santos IS, Matijasevich A, Tavares BF, Barros AJD, Botelho IP, Lapolli C, Magalhâes PVS, Barbosa APPNB, Barros FC (2007). Validation of the Edinburgh Postnatal Depression Scale EPDS in a sample of mothers from the 2004 Pelotas Birth Cohort Study. Cad Saúde Pública.

[34] Zimet GD, Dahlem NW, Zimet SG, Farley GK (1988). The multidimensional scale of perceived social support. J Pers Assessment.

[35] Carvalho S, Pinto-Gouveia J, Pimentel P, Maia D, Mota-Pereira J (2011). Características psicométricas da versão portuguesa da Escala Multidimensional de Suporte Social Percebido. Psychologica.

[36] Associação Brasileira de Empresas de Pesquisa: Critério de Classificação Econômica Brasil. ABEP Org. (2019). https://www.abep.org/criterio-brasil. Accessed 2 Nov 2022.

[37] LEGISWEB LTDA. Lei N*º* 20127 de 15/01/2020. 2023. https://www.legisweb.com.br/legislacao/?id=388956. Accessed 01 Jul 2023.

[38] Corrêa H, Malloy-Diniz L, Romano-Silva MA (2009). Patrícia Figueira Escala de Depressão Pós-Natal de Edimburgo Para Triagem No Sistema Público de Saúde Edinburgh Postnatal Depression Scale for Screening in the Public Health System. Rev Saúde Pública.

[39] Zimet G. Multidimensional Scale of Perceived Social Support (MSPSS)-Scale Items and Scoring Information. 2016. http://gzimet.wix.com/mspss. Accessed 10 Apr 2022.

[40] R Core Team R: A language and environment for statistical computing. R Foundation for Statistical Computing, Vienna, Austria. 2021. https://www.R-project.org/. Accessed 3 May 2022.

[41] O’Connell MA, Leahy-Warren P, Khashan AS, Kenny LC, O’Neill SM (2017). Worldwide prevalence of tocophobia in pregnant women: systematic review and meta-analysis. Acta Obstet Gynecol Scand.

[42] Rondung E, Thomtén J, Sundin Ö (2016). Psychological perspectives on fear of childbirth. J Anxiety Disord.

[43] Roosevelt LK, Low LK (2021). Understanding fear of childbirth among underrepresented populations in the United States. J Health Psychol.

[44] Størksen HT, Garthus-Niegel S, Adams SS, Vangen S, Eberhard-Gran M (2015). Fear of childbirth and elective caesarean section: a population-based study. BMC Pregnancy Childbirth.

[45] Tsui MH, Pang MW, Melender HL, Xu L, Lau TK, Leung TN (2006). Maternal fear associated with pregnancy and childbirth in Hong Kong Chinese women. Women Health.

[46] Melender HL (2002). Fears and coping strategies associated with pregnancy and childbirth in Finland. J Midwifery Womens Health.

[47] Rezende CB (2011). Um Estado Emotivo: Representação Da Gravidez Na Mídia. Cad Pagu.

[48] Pereira RR, Franco SC, Baldin N (2011). A dor e o protagonismo da mulher na parturição. Rev Bras Anestesiol.

[49] Nascimento KIM, de Souza Lima V, Novaes CDP (2021). Manobra de Kristeller: uma violência obstétrica. Braz J Health Rev.

[50] O’Connell MA, Leahy-Warren P, Kenny LC, O’Neill SM, Khashan AS (2019). The prevalence and risk factors of fear of childbirth among pregnant women: a cross-sectional study in Ireland. Acta Obstet Gynecol Scand.

[51] Heimstad R, Dahloe R, Laache I, Skogvoll E, Schei B (2006). Fear of childbirth and history of abuse: Implications for pregnancy and delivery. Acta Obstet Gynecol Scand.

[52] Essén B, Binder P, Johnsdotter S (2011). An anthropological analysis of the perspectives of Somali women in the West and their obstetric care providers on caesarean birth. J Psychosom Obstet Gynecol.

[53] Simonsen T, Wahl A, Vangen S, Eberhard-Gran M (2013). Do previous abortions cause fear of childbirth?. Tidsskr Nor laegeforen.

[54] Koc AE, Colak S, Colak GV, Pusuroglu M, Hocaoglu C (2021). Investigating fear of childbirth in pregnant women and its relationship between anxiety sensitivity and somatosensory amplification. J Obstet Gynaecol.

[55] Mortazavi F, Agah J (2018). Childbirth fear and associated factors in a sample of pregnant Iranian Women. Oman Med J.

[56] Mohamamdirizi S, Mohamadirizi M, Mohamadirizi S (2018). The comparison of fear of childbirth and sense of coherence among low-risk and high-risk pregnancy women. J Educ Health Promot.

[57] Taubman-Ben-Ari O, Chasson M, Abu-Sharkia S (2021). Childbirth anxieties in the shadow of COVID-19: self-compassion and social support among Jewish and Arab pregnant women in Israel. Health Soc Care Community.

[58] Onchonga D, MoghaddamHosseini V, Keraka M, Várnagy Á (2020). Prevalence of fear of childbirth in a sample of gravida women in Kenya. Sex Reprod Health.

[59] Paraná. Law nº 20127/2020, january 15, 2020. Diário Oficial do Paraná nº10605. Poder Executivo Estadual. Assembleia Legislativa do Estado do Paraná. https://www.assembleia.pr.leg.br/legislacao/constituicao-estadual. Accessed 2 Nov 2022.

[60] Dymecka J, Gerymski R, Iszczuk A, Bidzan M (2021). Fear of coronavirus, stress and fear of childbirth in polish pregnant women during the covid-19 pandemic. Int J Environ Res Public Health.

[61] Mappa I, Distefano FA, Rizzo G (2020). Effects of coronavirus 19 pandemic on maternal anxiety during pregnancy: a prospectic observational study. J Perinat Med.

[62] do Souto SPA, de Albuquerqu RS, Prata AP (2020). Fear of childbirth in time of the new coronavirus pandemic. Rev Bras Enferm.

[63] Nomura R, Tavares I, Ubinha AC (2021). Impact of the covid-19 pandemic on maternal anxiety in Brazil. J Clin Med.

