# Supplementary material for: Fear of childbirth: prevalence and associated factors in pregnant women of a maternity hospital in southern Brazil
Source: BMC Pregnancy Childbirth. 2023 Sep 2;23:632. doi: 10.1186/s12884-023-05948-0 (PMC10474709; doi:10.1186/s12884-023-05948-0)
Supplement: Supplementary file 1 — Addition file 1: Table 1. The Wijma Delivery Expectancy/Experience Questionnaire (W-DEQ) version A translated into Portuguese (Brazil) by the authors. Table 2. The Wijma Delivery Expectancy/Experience Questionnaire (W-DEQ) version A © 1996 K. Wijma & B. Wijma. [file 12884_2023_5948_MOESM1_ESM.docx]

| **Table 1** The Wijma Delivery Expectancy/Experience Questionnaire (W-DEQ) version A translated into Portuguese (Brazil) by the authors | | | |
| --- | --- | --- | --- |
| 1. Como você pensa que o seu trabalho de parto e parto será de fato como um todo? | | | |
| 1. | Extremamente fantástico | 1 2 3 4 5 6 | Nem um pouco fantástico |
| 2. | Extremamente assustador | 1 2 3 4 5 6 | Nem um pouco assustador |
| 1. Como você pensa que irá se sentir, de forma geral, no trabalho de parto e parto? | | | |
| 3. | Extremamente solitária | 1 2 3 4 5 6 | Nem um pouco solitária |
| 4. | Extremamente forte | 1 2 3 4 5 6 | Nem um pouco forte |
| 5. | Extremamente confiante | 1 2 3 4 5 6 | Nem um pouco confiante |
| 6. | Extremamente com medo | 1 2 3 4 5 6 | Nem um pouco com medo |
| 7. | Extremamente sozinha | 1 2 3 4 5 6 | Nem um pouco sozinha |
| 8. | Extremamente fraca | 1 2 3 4 5 6 | Nem um pouco fraca |
| 9. | Extremamente em segurança | 1 2 3 4 5 6 | Nem um pouco em segurança |
| 10. | Extremamente independente | 1 2 3 4 5 6 | Nem um pouco independente |
| 11. | Extremamente desolada | 1 2 3 4 5 6 | Nem um pouco desolada |
| 12. | Extremamente tensa | 1 2 3 4 5 6 | Nem um pouco tensa |
| 13. | Extremamente satisfeita | 1 2 3 4 5 6 | Nem um pouco satisfeita |
| 14. | Extremamente orgulhosa | 1 2 3 4 5 6 | Nem um pouco orgulhosa |
| 15. | Extremamente abandonada | 1 2 3 4 5 6 | Nem um pouco abandonada |
| 16. | Extremamente serena, no controle dos meus sentimentos | 1 2 3 4 5 6 | Nem um pouco serena, sem controle dos meus sentimentos |
| 17. | Extremamente relaxada | 1 2 3 4 5 6 | Nem um pouco relaxada |
| 18. | Extremamente feliz | 1 2 3 4 5 6 | Nem um pouco feliz |
| 1. Como você pensa que irá se sentir no trabalho de parto e parto? | | | |
| 19. | Extremamente em pânico | 1 2 3 4 5 6 | Nem um pouco em pânico |
| 20. | Extremamente em desesperança | 1 2 3 4 5 6 | Nem um pouco em desesperança |
| 21. | Extremamente ansiando pela criança | 1 2 3 4 5 6 | Nem um pouco ansiando pela criança |
| 22. | Extremamente com autoconfiança | 1 2 3 4 5 6 | Nem um pouco com autoconfiança |
| 23. | Extremamente confiando (nos outros) | 1 2 3 4 5 6 | Nem um pouco confiando (nos outros) |
| 24. | Extremamente com dor | 1 2 3 4 5 6 | Nem um pouco com dor |
| 1. O que você pensa que irá ocorrer no momento mais intenso do parto? | | | |
| 25. | Eu vou me comportar muito mal | 1 2 3 4 5 6 | Eu não vou me comportar nem um pouco mal |
| 26. | Terei coragem de renunciar o controle do meu corpo | 1 2 3 4 5 6 | Não terei coragem de renunciar o controle do meu corpo |
| 27. | Eu vou perder totalmente o controle de mim | 1 2 3 4 5 6 | Eu não vou perder nada o controle de mim |
| 1. Como você imagina que irá perceber o exato momento que o bebê nascer? | | | |
| 28. | Extremamente curioso/engraçado | 1 2 3 4 5 6 | Nem um pouco curioso/engraçado |
| 29. | Extremamente natural | 1 2 3 4 5 6 | Nem um pouco natural |
| 30. | Extremamente óbvio | 1 2 3 4 5 6 | Nem um pouco óbvio |
| 31. | Extremamente perigoso | 1 2 3 4 5 6 | Nem um pouco perigoso |
| 1. Você teve, durante o último mês, pensamentos sobre o trabalho de parto e parto, por exemplo... | | | |
| 32. | ... imaginou que seu filho irá morrer durante o parto? | | |
|  | Nunca | 1 2 3 4 5 6 | Muitas vezes |
| 33. | ... imaginou que seu filho será ferido durante o parto? | | |
|  | Nunca | 1 2 3 4 5 6 | Muitas vezes |

| **Table 2** The Wijma Delivery Expectancy/Experience Questionnaire (W-DEQ) version A © 1996 K. Wijma & B. Wijma | | | |
| --- | --- | --- | --- |
| 1. How do you think your labor and delivery will turn out as a whole? | | | |
| 1. | Extremely fantastic | 1 2 3 4 5 6 | Not at all fantastic |
| 2. | Extremely frightful | 1 2 3 4 5 6 | Not at all frightful |
| 1. How do you think you will feel in general during the labour and delivery? | | | |
| 3. | Extremely lonely | 1 2 3 4 5 6 | Not at all lonely |
| 4. | Extremely strong | 1 2 3 4 5 6 | Not at all strong |
| 5. | Extremely confident | 1 2 3 4 5 6 | Not at all confident |
| 6. | Extremely afraid | 1 2 3 4 5 6 | Not at all afraid |
| 7. | Extremely deserted | 1 2 3 4 5 6 | Not at all deserted |
| 8. | Extremely weak | 1 2 3 4 5 6 | Not at all weak |
| 9. | Extremely safe | 1 2 3 4 5 6 | Not at all safe |
| 10. | Extremely independent | 1 2 3 4 5 6 | Not at all independent |
| 11. | Extremely desolate | 1 2 3 4 5 6 | Not at all desolate |
| 12. | Extremely tense | 1 2 3 4 5 6 | Not at all tense |
| 13. | Extremely glad | 1 2 3 4 5 6 | Not at all glad |
| 14. | Extremely proud | 1 2 3 4 5 6 | Not at all proud |
| 15. | Extremely abandoned | 1 2 3 4 5 6 | Not at all abandoned |
| 16. | Totally composed | 1 2 3 4 5 6 | Not at all composed |
| 17. | Extremely relaxed | 1 2 3 4 5 6 | Not at al relaxedl |
| 18. | Extremely happy | 1 2 3 4 5 6 | Not at all happy |
| 1. How do you think you will feel during the labour and delivery? | | | |
| 19. | Extreme panic | 1 2 3 4 5 6 | No panic at all |
| 20. | Extreme hopelessness | 1 2 3 4 5 6 | No hopelessness at all |
| 21. | Extreme longing for the child | 1 2 3 4 5 6 | No longing for the child at all |
| 22. | Extreme self-confidence | 1 2 3 4 5 6 | No self-confidence at all |
| 23. | Extreme trust | 1 2 3 4 5 6 | No trust at all |
| 24. | Extreme pain | 1 2 3 4 5 6 | No pain at all |
| 1. What do you think will happen when labour is most intense? | | | |
| 25. | I will behave extremely badly | 1 2 3 4 5 6 | I will not behave badly at all |
| 26. | I will dare to totally surrender control to my body | 1 2 3 4 5 6 | I will not dare to surrender control to my body at all |
| 27. | I will totally lose control of myself | 1 2 3 4 5 6 | I will not lose control of myself at all |
| 1. How do you imagine it will feel the very moment you deliver the baby? | | | |
| 28. | Extremely funny | 1 2 3 4 5 6 | Not at all funny |
| 29. | Extremely natural | 1 2 3 4 5 6 | Not at all natural |
| 30. | Extremely self-evident | 1 2 3 4 5 6 | Not at all self-evident |
| 31. | Extremely dangerous | 1 2 3 4 5 6 | Not at all dangerous |
| 1. Have you, during the last month, had fantasies about the labour and delivery, for example… | | | |
| 32. | ... fantasies that your child will die during labour/delivery? | | |
|  | Never | 1 2 3 4 5 6 | Very often |
| 33. | ... fantasies that your child will be injured during labour/delivery? | | |
|  | Never | 1 2 3 4 5 6 | Very often |
